# Association of the Pan-Immune-Inflammation Value with Persistent Proteinuria in Patients with Familial Mediterranean Fever

**DOI:** 10.3390/jcm15218459

**Published:** 2026-10-30

**Authors:** Hasan Göğebakan, Ahmed Ramiz Baykan, Büşra Karadavut, Mekiye Damla Özdemir, Turgut Özdemir, Nizameddin Koca

**Affiliations:** 1 Division of Rheumatology, Department of Internal Medicine, Bursa City Hospital, University of Health Sciences, Bursa 16110, Turkey; 2 Division of Gastroenterology, Department of Internal Medicine, Erzurum Regional Training and Research Hospital, Erzurum 25240, Turkey; 3 Department of Internal Medicine, Bursa City Hospital, University of Health Sciences, Bursa 16110, Turkey

**Keywords:** familial mediterranean fever, M694V genotype, pan-immune-inflammation value, persistent proteinuria

## Abstract

**Background and Objectives:** Persistent proteinuria is an important marker of renal involvement in familial Mediterranean fever (FMF). This study investigated the association between the pan-immune-inflammation value (PIV) and persistent proteinuria, evaluated the discriminatory performance of PIV, the neutrophil-to-lymphocyte ratio (NLR), and monocyte-to-HDL cholesterol ratio (MHR), and identified factors independently associated with persistent proteinuria. **Materials and Methods:** This retrospective cross-sectional study included 190 adults with FMF and 150 healthy controls. Persistent proteinuria was defined as a spot urine protein-to-creatinine ratio (UPCR) > 20 mg/mmol confirmed on at least two measurements obtained at least 1 week apart. PIV quartile analysis, Spearman correlation, ROC analysis, and multivariable logistic regression were performed. **Results:** Persistent proteinuria was present in 62 (32.6%) patients with FMF. Its prevalence increased progressively across PIV quartiles (*p* for trend < 0.001), and PIV was positively correlated with UPCR (ρ = 0.298, *p* < 0.001). PIV showed an AUC of 0.774, compared with 0.658 for NLR and 0.654 for MHR. In multivariable analysis, PIV (OR 1.006, 95% CI 1.002–1.009, *p* = 0.001) and homozygous M694V genotype (OR 3.39, 95% CI 1.46–7.86, *p* = 0.004) were independently associated with persistent proteinuria. **Conclusions:** PIV and homozygous M694V genotype were independently associated with persistent proteinuria. PIV may serve as an adjunctive biomarker for the assessment of persistent proteinuria in patients with FMF, although prospective multicenter studies are needed to determine its clinical utility.

## 1. Introduction

Familial Mediterranean fever (FMF) is the most common monogenic autoinflammatory disease and is characterized by recurrent episodes of fever and serosal inflammation [1,2,3]. It results from pathogenic variants in the Mediterranean fever (*MEFV*) gene, which encodes pyrin, leading to dysregulated inflammasome activation and excessive interleukin-1β (IL-1β) production [1,2,3]. Although colchicine effectively controls inflammatory attacks and reduces disease-related complications, subclinical inflammation may persist during attack-free periods in a proportion of patients [2,3,4].

Renal involvement is an important complication of FMF and a major determinant of long-term morbidity. Amyloid A (AA) amyloidosis is the most severe renal complication of FMF; however, proteinuria may occur before clinically overt amyloidosis and is an important marker of renal involvement [5,6,7]. Persistent proteinuria was selected as the primary renal outcome because it is more likely to reflect sustained renal involvement, whereas transient proteinuria may occur in the context of acute inflammation or other reversible conditions [8]. Persistent inflammation and the homozygous M694V genotype have been associated with proteinuria and other renal complications in patients with FMF [6,7,9]. These findings highlight the importance of identifying markers that may reflect ongoing inflammatory activity and renal involvement in patients with FMF.

Inflammation-based hematological indices derived from routinely available laboratory parameters have been increasingly studied in FMF. The neutrophil-to-lymphocyte ratio (NLR) and monocyte-to-high-density lipoprotein cholesterol ratio (MHR) have been evaluated in patients with FMF and have been associated with inflammatory and disease-related features [10,11]. The pan-immune-inflammation value (PIV), which integrates neutrophil, monocyte, platelet, and lymphocyte counts, has also been investigated as a marker of systemic inflammation in FMF and other inflammatory conditions [12,13,14]. In FMF, higher PIV levels have been associated with disease severity and AA amyloidosis [14]. More recently, a study of 386 adults with FMF reported that PIV was independently associated with both *MEFV* mutation burden and AA amyloidosis [15], further supporting its potential relevance to inflammatory burden during attack-free periods.

Previous studies have evaluated PIV in relation to disease severity and AA amyloidosis in FMF, but these studies did not specifically assess persistent proteinuria as an outcome [14,15]. It is also unclear whether PIV provides additional information on persistent proteinuria beyond selected clinical, inflammatory, and genetic characteristics. In addition, data on the discriminatory performance of PIV in relation to other readily available inflammation-based indices, such as NLR and MHR, are limited. Accordingly, this study investigated the association between PIV and persistent proteinuria in patients with FMF, evaluated the discriminatory performance of PIV, NLR, and MHR, and assessed factors independently associated with persistent proteinuria.

## 2. Materials and Methods

### 2.1. Study Design and Participants

This retrospective cross-sectional study was conducted at the Department of Rheumatology, Bursa City Hospital, a tertiary referral center, between December 2023 and December 2025. Consecutive adults (≥18 years) with familial Mediterranean fever (FMF) diagnosed according to the Tel Hashomer criteria [16] were identified through the hospital electronic medical record system. To minimize the potential influence of acute inflammatory activity, only patients evaluated during attack-free periods, defined as at least two weeks after the last FMF attack, were included.

Patients were identified through the hospital electronic medical record system and were evaluated during outpatient follow-up visits; the study was not restricted to patients assessed at the time of FMF diagnosis. The interval between symptom onset and FMF diagnosis was not systematically available in the retrospective medical records and therefore could not be reliably analyzed.

Patients with diabetes mellitus, hypertension, chronic kidney disease, active infection, malignancy, pregnancy, concomitant inflammatory or autoimmune diseases, or other conditions that could affect inflammatory status, hematological parameters, or serum high-density lipoprotein cholesterol (HDL-C) levels were excluded. Patients receiving medications known to substantially affect inflammatory markers, hematological parameters, or HDL-C levels were also excluded. Of the 205 patients with FMF initially evaluated for eligibility, 15 patients were excluded because of incomplete demographic, clinical, laboratory, genetic, or urinary data. The final study population therefore consisted of 190 patients with FMF.

The control group comprised healthy adults recruited from the hospital health screening clinic during the same study period and frequency-matched by age and sex. Controls had no history of inflammatory, autoimmune, infectious, malignant, renal, hepatic, cardiovascular, or metabolic diseases and were not receiving medications known to influence inflammatory status, hematological parameters, or HDL-C levels.

The study was designed and reported in accordance with the Strengthening the Reporting of Observational Studies in Epidemiology (STROBE) guidelines [17].

### 2.2. Ethics Statement

The study protocol was approved by the Bursa City Hospital Scientific Research Ethics Committee (Decision No. 2026-08/3; 15 April 2026). The study was conducted in accordance with the ethical principles of the Declaration of Helsinki.

### 2.3. Data Collection and Definitions

Demographic characteristics, disease duration, laboratory parameters, urinary findings, and *MEFV* genotypes were retrieved from the hospital electronic medical record system. Venous blood and spot urine samples were obtained during outpatient visits conducted during attack-free periods. Laboratory assessments included complete blood count, C-reactive protein (CRP), erythrocyte sedimentation rate (ESR), serum amyloid A (SAA), creatinine, and high-density lipoprotein cholesterol (HDL-C). The blood samples used to calculate PIV were obtained during the same outpatient assessment as the other laboratory measurements. The same laboratory analyzers were used throughout the study period, with no changes in the analytical platforms during the study. Complete blood count parameters, including neutrophil, lymphocyte, monocyte, and platelet counts, were measured using an automated hematology analyzer (Sysmex XN-9100, Sysmex Corporation, Kobe, Japan). CRP concentrations were measured using an immunoturbidimetric method on a Roche Cobas c703 analyzer (Roche Diagnostics, Mannheim, Germany). SAA concentrations were measured by nephelometry using a BN nephelometric analyzer (Siemens Healthineers, Erlangen, Germany).

Treatment status at the time of inclusion was also recorded, including colchicine and interleukin-1 (IL-1) inhibitor use. IL-1 inhibitor treatment included anakinra or canakinumab.

Proteinuria was defined as a spot urine protein-to-creatinine ratio (UPCR) >20 mg/mmol [7]. Persistent proteinuria was defined as a UPCR >20 mg/mmol confirmed on at least two measurements obtained at least 1 week apart. Patients were classified according to the presence or absence of persistent proteinuria.

*MEFV* genotypes were recorded for all patients, with particular attention to homozygous M694V status. The neutrophil-to-lymphocyte ratio (NLR) was calculated as the neutrophil count divided by the lymphocyte count, and the monocyte-to-high-density lipoprotein cholesterol ratio (MHR) was calculated as the monocyte count divided by the HDL-C level [10,11]. The pan-immune-inflammation value (PIV) was calculated according to the previously described formula [12]:

PIV = (neutrophil count × platelet count × monocyte count)/lymphocyte count, with all cell counts expressed as ×10^3^/µL.

### 2.4. Statistical Analysis

Statistical analyses were performed using IBM SPSS Statistics for Windows, version 27.0 (IBM Corp., Armonk, NY, USA). The distribution of continuous variables was assessed using histograms, probability plots, and the Kolmogorov–Smirnov and Shapiro–Wilk tests. Continuous variables are presented as median (interquartile range [IQR]), whereas categorical variables are presented as frequencies and percentages.

Comparisons between patients with FMF and healthy controls and between patients with and without persistent proteinuria were performed using the Mann–Whitney U test for continuous variables and the chi-square (χ^2^) test or Fisher’s exact test for categorical variables, as appropriate.

Spearman’s rank correlation analysis was used to assess the associations of PIV, NLR, and MHR with clinical and laboratory variables. Patients were categorized into quartiles according to PIVs, and the prevalence of persistent proteinuria across PIV quartiles was assessed using the chi-square test for trend.

Receiver operating characteristic (ROC) curve analysis was performed to assess the discriminatory performance of PIV, NLR, and MHR for identifying persistent proteinuria. The area under the curve (AUC) with 95% confidence intervals (CIs), optimal cut-off values determined using the Youden index, sensitivity, and specificity were calculated. Pairwise comparisons of ROC AUCs were performed using DeLong’s test for correlated ROC curves.

Univariable and multivariable logistic regression analyses were performed to identify factors associated with persistent proteinuria. Variables were selected based on clinical relevance and previous literature, including age, sex, homozygous M694V genotype, SAA, NLR, MHR, and PIV, and were entered simultaneously into the multivariable model. Because MHR values were numerically small, MHR was rescaled per 0.01-unit increase to facilitate interpretation of the odds ratio. Multicollinearity among predictors was assessed using variance inflation factors (VIFs). No substantial linear multicollinearity was identified based on the VIF values. Odds ratios (ORs) with 95% CIs were reported. A two-sided *p* value < 0.05 was considered statistically significant.

## 3. Results

### 3.1. Baseline Characteristics

Baseline demographic, laboratory, and inflammatory biomarker characteristics of the study population are presented in Table 1. Patients with FMF and healthy controls were comparable in terms of age (*p* = 0.254) and sex (*p* = 0.910). Compared with healthy controls, patients with FMF had higher neutrophil (*p* = 0.014), monocyte (*p* = 0.023), and platelet counts (*p* < 0.001), as well as higher CRP, NLR, MHR, and PIV levels (all *p* < 0.001). Lymphocyte counts (*p* = 0.046) and HDL-C levels (*p* < 0.001) were lower in patients with FMF. No significant differences were observed in white blood cell count (*p* = 0.171), hemoglobin (*p* = 0.238), ESR (*p* = 0.102), or creatinine levels (*p* = 0.921) between the two groups.

### 3.2. Persistent Proteinuria and Inflammatory Biomarkers

Among the 190 patients with FMF, 62 (32.6%) had persistent proteinuria. Patients with persistent proteinuria had higher CRP, ESR, SAA, NLR, MHR, and PIV levels and lower HDL-C levels than those without persistent proteinuria (all *p* ≤ 0.002). Among the 62 patients with persistent proteinuria, 26 (41.9%) had CRP and SAA levels within the normal reference ranges. Of these, 10 (38.5%) had a PIV above the ROC-derived cut-off of 352.48, corresponding to 16.1% of all patients with persistent proteinuria. Pairwise comparison using DeLong’s test showed that the AUC for PIV was not significantly different from that for NLR (*p* = 0.142) or MHR (*p* = 0.342). Homozygous M694V was more frequent among patients with persistent proteinuria than among those without persistent proteinuria (50.0% vs. 22.7%, *p* < 0.001). Age, sex, disease duration, and creatinine levels did not differ significantly between the two groups (Table 2). At study inclusion, 187 (98.4%) patients were receiving colchicine, and 35 (18.4%) were receiving an interleukin-1 (IL-1) inhibitor; among patients receiving IL-1 inhibition, treatment consisted of anakinra or canakinumab.

**Figure 1:**
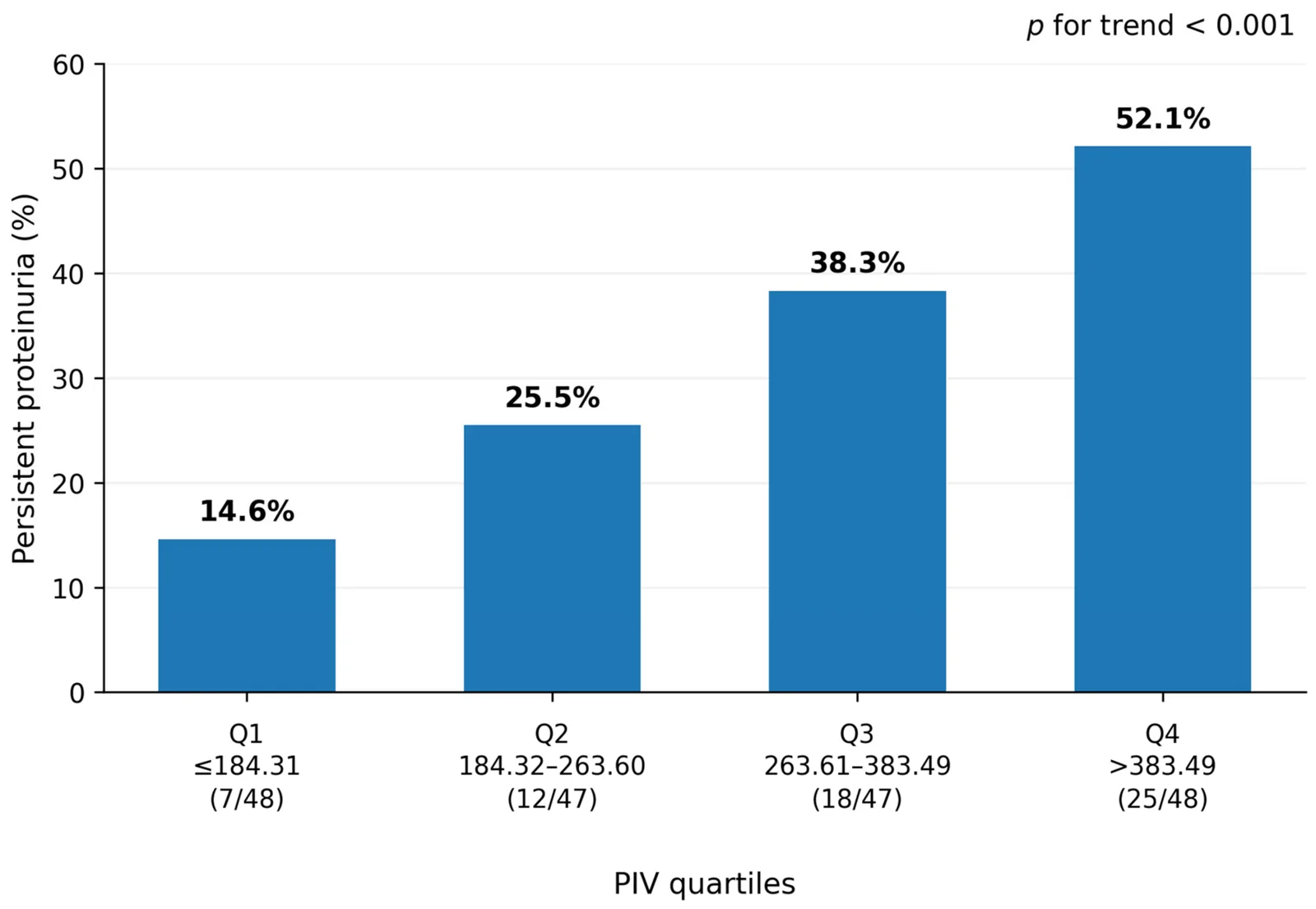
Prevalence of persistent proteinuria across PIV quartiles in patients with familial Mediterranean fever. Data are shown as percentages; numbers in parentheses indicate patients with persistent proteinuria/total patients in each quartile. *p* for trend < 0.001.

The prevalence of persistent proteinuria increased progressively across PIV quartiles, from 14.6% in the lowest quartile to 52.1% in the highest quartile (*p* for trend < 0.001) (Figure 1).

### 3.3. ROC Curve Analysis

**Figure 2:**
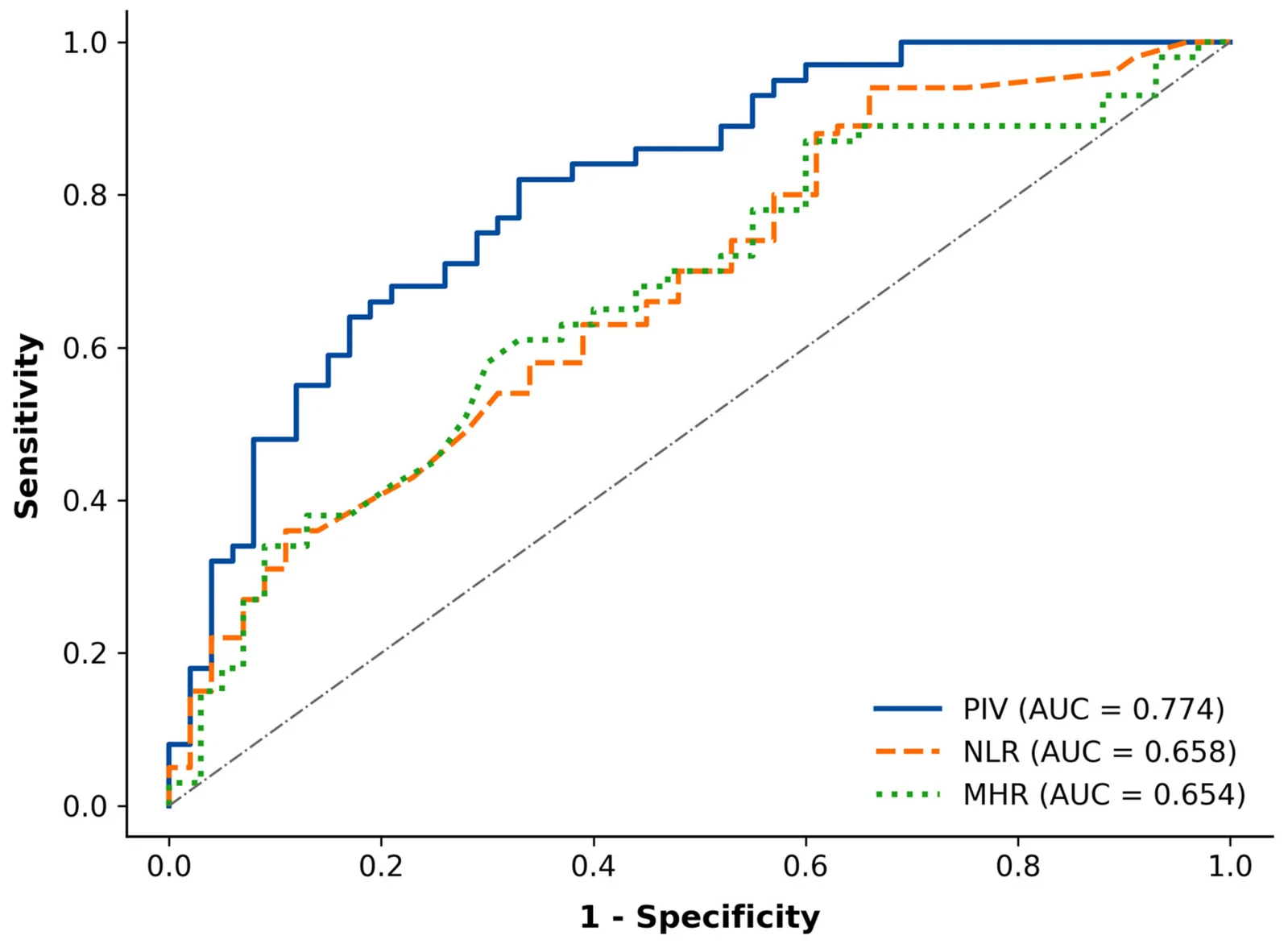
Receiver operating characteristic (ROC) curves of pan-immune-inflammation value (PIV), neutrophil-to-lymphocyte ratio (NLR), and monocyte-to-HDL cholesterol ratio (MHR) for identifying persistent proteinuria in patients with familial Mediterranean fever.

The results of the receiver operating characteristic (ROC) curve analysis are presented in Table 3 and Figure 2. PIV showed an area under the curve (AUC) of 0.774 (95% CI, 0.689–0.850) for identifying persistent proteinuria. The corresponding AUCs were 0.658 (95% CI, 0.559–0.742) for NLR and 0.654 (95% CI, 0.554–0.745) for MHR.

The optimal PIV cut-off value was 352.48, with a sensitivity of 65.9% and a specificity of 78.8%. Optimal cut-off values were determined using the Youden index.

### 3.4. Correlation and Multivariable Analysis

PIV was positively correlated with the spot UPCR (ρ = 0.298, *p* < 0.001), CRP (ρ = 0.327, *p* < 0.001), ESR (ρ = 0.246, *p* < 0.001), and SAA (ρ = 0.297, *p* < 0.001). NLR was also positively correlated with the spot UPCR (ρ = 0.196, *p* < 0.01), CRP (ρ = 0.339, *p* < 0.001), and SAA (ρ = 0.213, *p* < 0.01). MHR showed weaker positive correlations with the spot UPCR (ρ = 0.144, *p* < 0.05), ESR (ρ = 0.165, *p* < 0.05), and SAA (ρ = 0.193, *p* < 0.01), and was negatively correlated with HDL-C (ρ = −0.486, *p* < 0.001) (Table 4).

In the multivariable logistic regression analysis, PIV remained independently associated with persistent proteinuria (OR 1.006, 95% CI, 1.002–1.009; *p* = 0.001), as did the homozygous M694V genotype (OR 3.39, 95% CI, 1.46–7.86; *p* = 0.004). Age, sex, SAA, NLR, and MHR were not independently associated with persistent proteinuria after adjustment for the other variables in the model (Table 5). No substantial linear multicollinearity was identified based on the VIF values.

## 4. Discussion

In this study, PIV was significantly higher in FMF patients with persistent proteinuria than in those without persistent proteinuria. PIV was positively correlated with the spot urine protein-to-creatinine ratio, and the prevalence of persistent proteinuria increased progressively across PIV quartiles. In ROC curve analysis, PIV showed an AUC of 0.774 for identifying persistent proteinuria, compared with 0.658 for NLR and 0.654 for MHR. In multivariable logistic regression analysis, both PIV and homozygous M694V genotype were independently associated with persistent proteinuria.

PIV combines neutrophil, monocyte, platelet, and lymphocyte counts into a single index and was initially described as a marker of systemic inflammation [12]. Neutrophils may contribute to renal injury through oxidative stress, proteolytic activity, and the formation of neutrophil extracellular traps, potentially promoting endothelial and glomerular damage [18]. Monocytes may further sustain renal inflammation through tissue recruitment and differentiation into macrophages, and platelets may participate in inflammatory responses through platelet–leukocyte interactions and endothelial activation [19,20]. Lymphocytes have more context-dependent effects, as different lymphocyte subsets may either promote renal inflammation or contribute to its resolution [21]. Collectively, these mechanisms provide biological plausibility for the observed association between increased PIV and renal involvement in FMF. However, the present study cannot establish causality or determine the direct contribution of individual cellular components to persistent proteinuria.

Its association with clinical outcomes has subsequently been investigated in population-based studies and various inflammatory diseases [13,22,23,24,25]. In FMF, Ocak et al. reported that higher PIV levels were associated with disease severity and AA amyloidosis [14]. More recently, a larger cohort of 386 adults with FMF demonstrated independent associations of PIV with *MEFV* mutation burden and AA amyloidosis [15]. Our findings further support an association between PIV and persistent proteinuria in this cohort, a clinically relevant marker of renal involvement. This association remained significant after adjustment for SAA, NLR, MHR, age, sex, and homozygous M694V genotype. PIV may therefore reflect the inflammatory milieu associated with persistent proteinuria during attack-free periods. The observation that some patients with persistent proteinuria had normal CRP and SAA levels but elevated PIV suggests that PIV may provide complementary information beyond conventional acute-phase markers. However, whether PIV provides incremental predictive value beyond SAA has not been established in this study. Therefore, PIV should be considered a potential adjunctive marker rather than a replacement for SAA or urinary protein assessment.

Renal manifestations in FMF have been investigated in relation to systemic inflammation and disease characteristics. Proteinuria may precede clinically overt AA amyloidosis and is therefore an important marker of renal involvement in FMF [5,7]. Previous studies have also linked hematological inflammatory markers with renal manifestations; Sakallı and Kal reported an association between increased mean platelet volume and proteinuria and amyloidosis [26]. In our cohort, the prevalence of persistent proteinuria increased progressively across PIV quartiles, from 14.6% in the lowest quartile to 52.1% in the highest quartile. This graded pattern further supports the association between higher PIV levels and persistent proteinuria in patients with FMF.

The association between PIV and persistent proteinuria may be related, at least in part, to the chronic inflammatory state characteristic of FMF. PIV incorporates neutrophil, monocyte, platelet, and lymphocyte counts and therefore reflects several cellular components of the inflammatory response. Subclinical inflammation may persist during attack-free periods in patients with FMF as a result of ongoing inflammasome activation and interleukin-1β production [1,2,3]. Persistent inflammatory activity has been implicated in endothelial dysfunction and renal injury and may contribute to amyloid deposition and proteinuria [5,27]. This association may partly reflect the link between systemic inflammatory activity and renal involvement in FMF.

Previous studies have used ROC analysis to assess the discriminatory performance of PIV in FMF and other inflammatory diseases [14,23]. In our study, PIV showed an AUC of 0.774 (95% CI, 0.689–0.850) for identifying persistent proteinuria, compared with 0.658 (95% CI, 0.559–0.742) for NLR and 0.654 (95% CI, 0.554–0.745) for MHR. Although the AUC was numerically higher for PIV than for NLR and MHR, the differences were not statistically significant on pairwise comparison using DeLong’s test (PIV vs. NLR, *p* = 0.142; PIV vs. MHR, *p* = 0.342). Nevertheless, the observed AUC suggests that PIV had moderate discriminatory ability for persistent proteinuria in this cohort.

SAA is an established marker of inflammatory activity in FMF and plays a central role in the pathogenesis of AA amyloidosis [5]. In our cohort, SAA levels were higher in patients with persistent proteinuria, but SAA was not independently associated with persistent proteinuria after adjustment for the other variables in the multivariable model. Subclinical inflammation may persist during attack-free periods and may not be fully reflected by individual acute-phase reactants [28,29]. PIV remained independently associated with persistent proteinuria after adjustment for SAA, suggesting that the observed association between PIV and persistent proteinuria was not explained by SAA alone.

The homozygous M694V genotype was also independently associated with persistent proteinuria in our cohort. This finding is consistent with previous reports associating M694V homozygosity with a more severe FMF phenotype [9,30] and with renal involvement, including proteinuria [6,7,31,32]. The association between M694V homozygosity and persistent proteinuria remained significant after adjustment for PIV and other clinical and inflammatory variables. This suggests that the association of M694V homozygosity with persistent proteinuria was not fully accounted for by the inflammatory markers included in the multivariable model.

### Strengths and Limitations

This study has several limitations. First, its retrospective cross-sectional design precludes causal inference. Second, the single-center setting and relatively modest sample size may limit the generalizability of the findings. Third, the absence of renal biopsy, minor salivary gland biopsy data, and standardized longitudinal follow-up data limited assessment of the underlying cause and clinical course of persistent proteinuria. In particular, histologically confirmed AA amyloid deposition could not be systematically assessed, and the available data did not allow us to determine whether PIV levels preceded the development of persistent proteinuria or amyloid deposition, or whether higher PIV was associated with subsequent progression to AA amyloidosis. Fourth, the number of patients with persistent proteinuria (*n* = 62) relative to the seven predictors included in the multivariable model resulted in approximately 8.9 events per variable (EPV), which is slightly below the commonly cited rule-of-thumb of 10 events per variable. This relatively low EPV may have increased the risk of model overfitting and reduced the precision and generalizability of the estimates. Although no substantial linear multicollinearity was identified based on VIF assessment, this does not eliminate the potential for overfitting. Furthermore, because PIV incorporates cellular components that are also included in the calculation of NLR and MHR, some degree of biological and mathematical overlap among these indices cannot be entirely excluded, despite the absence of substantial linear multicollinearity based on VIF assessment. Finally, although pairwise comparisons of the ROC curves were performed using DeLong’s test, the differences in AUCs between PIV and NLR or MHR were not statistically significant; therefore, the present ROC analysis did not demonstrate a statistically significant superiority of PIV over these biomarkers. In addition, residual confounding, including potential treatment-related confounding, cannot be excluded because treatment variables were not included in the multivariable model.

Despite these limitations, the study has several strengths. Persistent proteinuria was defined using a UPCR threshold >20 mg/mmol and required confirmation on at least two measurements obtained at least 1 week apart, reducing the likelihood that transient proteinuria was classified as persistent proteinuria. In addition, patients were evaluated during attack-free periods, allowing the association of PIV with persistent proteinuria to be examined outside an acute FMF attack. The study also evaluated PIV in relation to other inflammation-based indices and incorporated both inflammatory and genetic variables into the multivariable analysis.

PIV may be a useful adjunctive biomarker for identifying patients with persistent proteinuria in FMF. It is calculated from routinely available complete blood count parameters and does not require additional laboratory testing. Notably, 16.1% of patients with persistent proteinuria had normal CRP and SAA levels but an elevated PIV, suggesting that PIV may provide complementary information in a subset of patients in whom conventional inflammatory markers are not elevated. However, PIV should not be considered a substitute for urinary assessment or established clinical and laboratory measures, which remain essential for the evaluation of renal involvement. Rather, PIV may provide complementary information regarding systemic inflammatory burden in patients with FMF and renal involvement. The clinical utility of this complementary role remains to be established in prospective longitudinal and multicenter studies.

## 5. Conclusions

PIV and homozygous M694V genotype were independently associated with persistent proteinuria in patients with FMF. PIV demonstrated moderate discriminatory ability for persistent proteinuria and may serve as an adjunctive biomarker. Prospective multicenter studies are needed to validate these findings and determine the clinical utility of PIV.

## Figures and Tables

**Table 1:** Baseline demographic, laboratory, and inflammatory biomarker characteristics of patients with familial Mediterranean fever and healthy controls.

Variable	FMF (*n* = 190)	Healthy Controls (*n* = 150)	*p*-Value
**Demographic characteristics**			
Age, years	33.5 (26.0–43.8)	36.0 (26.0–47.0)	0.254
Female sex, *n* (%)	110 (57.9)	86 (57.3)	0.910
**Laboratory characteristics**			
White blood cell count (×10^3^/µL)	7.58 (6.20–8.78)	7.24 (6.02–8.60)	0.171
Neutrophils (×10^3^/µL)	4.50 (3.44–5.40)	3.98 (3.19–4.83)	0.014
Lymphocytes (×10^3^/µL)	2.20 (1.87–2.68)	2.32 (1.99–2.85)	0.046
Monocytes (×10^3^/µL)	0.54 (0.43–0.65)	0.50 (0.40–0.59)	0.023
Hemoglobin (g/dL)	13.75 (12.50–14.90)	13.70 (12.70–15.17)	0.238
Platelets (×10^3^/µL)	258 (224–296)	230 (197–274)	<0.001
Creatinine (mg/dL)	0.71 (0.61–0.84)	0.71 (0.64–0.82)	0.921
CRP (mg/L)	3.36 (1.33–8.50)	1.20 (0.60–3.50)	<0.001
ESR (mm/h)	8.5 (3.0–13.0)	8.0 (6.0–12.0)	0.102
HDL-C levels (mg/dL)	44 (38.3–49.0)	48 (42.0–57.5)	<0.001
**Inflammatory biomarkers**			
Neutrophil-to-lymphocyte ratio (NLR)	1.94 (1.47–2.54)	1.62 (1.33–2.04)	<0.001
Monocyte-to-HDL cholesterol ratio (MHR)	0.0120 (0.0096–0.0158)	0.0102 (0.0078–0.0135)	<0.001
Pan-immune-inflammation value (PIV)	263.60 (184.31–383.49)	185.17 (135.12–250.43)	<0.001

Data are presented as median (interquartile range [IQR]) or *n* (%). CRP, C-reactive protein; ESR, erythrocyte sedimentation rate; FMF, familial Mediterranean fever; HDL-C, high-density lipoprotein cholesterol; IQR, interquartile range; MHR, monocyte-to-HDL cholesterol ratio; NLR, neutrophil-to-lymphocyte ratio; PIV, pan-immune-inflammation value.

**Table 2:** Clinical, laboratory, genetic, treatment, and inflammatory biomarker characteristics of patients with FMF according to the presence of persistent proteinuria.

Variable	Persistent Proteinuria (+) (*n* = 62)	Persistent Proteinuria (−) (*n* = 128)	*p*-Value
**Demographic characteristics**			
Age, years	37.0 (28.0–42.0)	32.0 (25.3–44.0)	0.269
Female sex, *n* (%)	35 (56.4)	75 (58.5)	0.870
Disease duration, years	13.5 (5.0–20.0)	11.0 (5.0–17.8)	0.431
**Genetic characteristics**			
Homozygous M694V, *n* (%)	31 (50.0)	29 (22.7)	<0.001
**Laboratory characteristics**			
Creatinine (mg/dL)	0.76 (0.65–0.85)	0.70 (0.60–0.84)	0.159
CRP (mg/L)	7.25 (2.91–12.70)	2.58 (1.07–6.17)	<0.001
ESR (mm/h)	13.0 (10.0–15.0)	6.0 (3.0–11.8)	<0.001
SAA (mg/L)	1.38 (0.84–6.60)	0.69 (0.36–1.92)	0.001
HDL-C levels (mg/dL)	39.5 (34.8–44.3)	45.0 (40.0–50.0)	<0.001
**Treatment characteristics**			
Colchicine use, *n* (%)	60 (96.8)	127 (99.2)	0.249
IL-1 inhibitor use, *n* (%)	15 (24.2)	20 (15.6)	0.166
**Inflammatory biomarkers**			
NLR	2.29 (1.75–2.99)	1.83 (1.41–2.44)	0.002
MHR	0.0138 (0.0096–0.0178)	0.0118 (0.0096–0.0154)	0.002
PIV	402.66 (281.65–567.50)	240.12 (176.52–322.70)	<0.001

Data are presented as median (interquartile range [IQR]) or *n* (%). CRP, C-reactive protein; ESR, erythrocyte sedimentation rate; FMF, familial Mediterranean fever; HDL-C, high-density lipoprotein cholesterol; IQR, interquartile range; IL-1, interleukin-1; MHR, monocyte-to-HDL cholesterol ratio; NLR, neutrophil-to-lymphocyte ratio; PIV, pan-immune-inflammation value; SAA, serum amyloid A.

**Table 3:** Receiver operating characteristic analysis of inflammatory biomarkers for identifying persistent proteinuria in patients with familial Mediterranean fever.

Biomarker	AUC (95% CI)	Cut-Off	Sensitivity (%)	Specificity (%)	*p*-Value
Pan-immune-inflammation value (PIV)	0.774 (0.689–0.850)	352.48	65.9	78.8	<0.001
Neutrophil-to-lymphocyte ratio (NLR)	0.658 (0.559–0.742)	1.65	86.4	39.0	0.003
Monocyte-to-HDL cholesterol ratio (MHR)	0.654 (0.554–0.745)	0.0136	56.8	69.9	0.004

*p* values indicate whether the AUC differed significantly from 0.50. Optimal cut-off values were determined using the Youden index. AUC, area under the curve; CI, confidence interval; HDL-C, high-density lipoprotein cholesterol; MHR, monocyte-to-HDL cholesterol ratio; NLR, neutrophil-to-lymphocyte ratio; PIV, pan-immune-inflammation value.

**Table 4:** Spearman correlation analysis between inflammatory biomarkers and clinical/laboratory parameters in patients with familial Mediterranean fever.

Biomarker	Spot Urine Protein-to-Creatinine Ratio (UPCR) (ρ)	CRP (ρ)	ESR (ρ)	SAA (ρ)	HDL-C (ρ)
Pan-immune-inflammation value (PIV)	0.298 ***	0.327 ***	0.246 ***	0.297 ***	−0.048
Neutrophil-to-lymphocyte ratio (NLR)	0.196 **	0.339 ***	0.140	0.213 **	−0.022
Monocyte-to-HDL cholesterol ratio (MHR)	0.144 *	0.135	0.165 *	0.193 **	−0.486 ***

Data are presented as Spearman’s correlation coefficients (ρ). * *p* < 0.05; ** *p* < 0.01; *** *p* < 0.001. CRP, C-reactive protein; ESR, erythrocyte sedimentation rate; HDL-C, high-density lipoprotein cholesterol; MHR, monocyte-to-high-density lipoprotein cholesterol ratio; NLR, neutrophil-to-lymphocyte ratio; PIV, pan-immune-inflammation value; SAA, serum amyloid A.

**Table 5:** Univariable and multivariable logistic regression analyses for factors associated with persistent proteinuria in patients with familial Mediterranean fever.

Variable	Univariable OR (95% CI)	*p*	Multivariable OR (95% CI)	*p*
Age (years)	1.02 (0.99–1.04)	0.282	1.03 (1.00–1.07)	0.062
Female sex	0.94 (0.48–1.87)	0.869	1.40 (0.60–3.25)	0.439
Homozygous M694V genotype	4.03 (1.97–8.27)	<0.001	3.39 (1.46–7.86)	0.004
Serum amyloid A (SAA)	1.003 (0.990–1.017)	0.624	1.004 (0.988–1.020)	0.635
Neutrophil-to-lymphocyte ratio (NLR)	1.37 (1.07–1.74)	0.012	0.75 (0.45–1.27)	0.287
Monocyte-to-HDL cholesterol ratio (MHR) *	3.56 (1.67–7.58)	0.001	1.64 (0.56–4.75)	0.366
Pan-immune-inflammation value (PIV)	1.005 (1.003–1.007)	<0.001	1.006 (1.002–1.009)	0.001

* MHR was entered into the regression model per 0.01-unit increase to improve interpretability of the estimated odds ratio. CI, confidence interval; HDL-C, high-density lipoprotein cholesterol; MHR, monocyte-to-HDL cholesterol ratio; NLR, neutrophil-to-lymphocyte ratio; OR, odds ratio; PIV, pan-immune-inflammation value; SAA, serum amyloid A.

## Data Availability

The datasets generated and/or analyzed during the current study are available from the corresponding author on reasonable request.

## Abbreviations

AA, amyloid A; AUC, area under the curve; CI, confidence interval; CRP, C-reactive protein; ESR, erythrocyte sedimentation rate; FMF, familial Mediterranean fever; HDL-C, high-density lipoprotein cholesterol; IL-1β, interleukin-1β; IQR, interquartile range; *MEFV*, Mediterranean fever gene; MHR, monocyte-to-HDL cholesterol ratio; NLR, neutrophil-to-lymphocyte ratio; OR, odds ratio; PIV, pan-immune-inflammation value; ROC, receiver operating characteristic; SAA, serum amyloid A; UPCR, urine protein-to-creatinine ratio; VIF, variance inflation factor.
